# Post-operative spread of small cell glioblastoma to the subcutaneous tissue via craniotomy defect in a 9-year-old boy: An exceptionally rare case report and review of the literature

**DOI:** 10.1016/j.ijscr.2025.111094

**Published:** 2025-02-25

**Authors:** Nooshin Zaresharifi, Anita Khalili, Behrad Eftekhari, Zoheir Reihanian

**Affiliations:** a Neuroscience Research Center, School of Medicine, Guilan University of Medical Sciences, Rasht, Iran; b Department of Medicine, Guilan University of Medical Sciences (GUMS), Rasht, Guilan, Iran

**Keywords:** Small cell glioblastoma, Glioblastoma, Craniotomy, Radiotherapy, Recurrent tumor invasion

## Abstract

**Introduction and importance:**

Glioblastoma multiforme (GBM), a grade 4 astrocytoma, represents a predominant malignant primary tumor within the central nervous system (CNS) and is known for its dismal prognosis. While extremely uncommon in pediatric cases, the small cell glioblastoma (scGBM) variant poses diagnostic challenges due to overlapping histopathological features with other intracranial tumors.

**Case presentation:**

We present the case of a 9-year-old Persian boy who underwent a craniotomy due to a malignant brain tumor. The histopathological examination, followed by ancillary immunohistochemical and molecular studies, led to the rare diagnosis of scGBM. Following a course of 32-session adjuvant radiotherapy, the tumor recurred with a peculiar manifestation, a subcutaneous mass at the previous surgical site, as a result of recurrent tumor invasion through craniotomy defect.

**Clinical discussion:**

Small-cell glioblastoma (scGBM) presents aggressive features, often misdiagnosed as other tumors. A rare pediatric occurrence, scGBM requires careful histopathological and molecular evaluations. Treatment aligns with classic GBM protocols, but prognosis is poor, with overall survival ranging from 5 to 23 months.

**Conclusion:**

The case highlights the diagnostic intricacies in distinguishing scGBM from other morphologically similar intracranial tumors, especially in pediatric patients. The report contributes to the limited literature on scGBM in the young population, emphasizing the significance of refined diagnostic approaches for accurate diagnosis and appropriate succeeding treatment for the best patient result.

## Highlights

• This report details a rare presentation of small cell glioblastoma (scGBM) in a 9-year-old boy.
• Similar histopathological featuresto other brain tumors underscores the mandatory need for immunohistochemical and molecular studies for definitive diagnosis in such cases.
• Post-radiotherapy, the tumor recurred as a subcutaneous mass at the craniotomy site, emphasizing the need for vigilance monitoring and the drastic aggressive nature of scGBM.

## 1 Introduction

Glioblastoma, a prevailing malignant primary tumor within the central nervous system (CNS), typically involves a poor prognosis, boasting an average survival of approximately one year [1]. The incidence of the high-grade histopathological variant, Glioblastoma multiforme (GBM), in the pediatric population is notably infrequent [2]. Furthermore, pediatric CNS tumors tend to predominantly originate within the posterior fossa, unlike their adult counterparts, which typically manifest in the supra-tentorial region [3].

Small cell glioblastoma (scGBM), classified as a rare histopathological subtype of the classical GBM, constitutes a minority, representing less than 10 % of all GBMs [4,5].

Small cell glioblastoma (SCGBM) is an aggressive brain tumor with a mean survival of less than one year. Its diagnosis is challenging due to similarities with classical glioblastoma multiforme (GBM) and other tumors like anaplastic oligodendroglioma and malignant lymphoma. Accurate identification requires thorough histopathological examination, immunohistochemistry (IHC) staining, and cytogenetic evaluations. Key diagnostic features include small, round to elongated astrocytes with a high nucleus-to-cytoplasmic (N:C) ratio, elevated mitotic activity, epidermal growth factor receptor (EGFR) amplification, and isocitrate dehydrogenase (IDH) wild-type status [5,6].This report outlines the clinical presentation and provides insights into the histopathological features, alongside IHC findings, explaining the diagnostic course of a 9-year-old child, culminating in the diagnosis of scGBM. This case has been reported in line with SCARE criteria [7].

## 2 Case presentation

A 9-year-old Persian boy was admitted to our emergency department with complaints of nausea, vomiting, and constant headache. Nausea and vomiting were initiated seven days prior and were resistant to anti-emetic agents. He also reported a 3-week history of dull, persistent, positional headaches on the left side of his skull. His past medical and medication history were unremarkable. Upon initial physical examination, the patient was alert, exhibiting a Glasgow Coma Scale (GCS) score of 15, with vital signs within the normal range. Pupillary examination revealed equal and reactive responses to light stimulation. Neurological assessments demonstrated intact cranial nerves, deep tendon reflexes graded at 2+, and symmetrical muscle strength was 5/5 across all limbs.

**Fig. 1:**
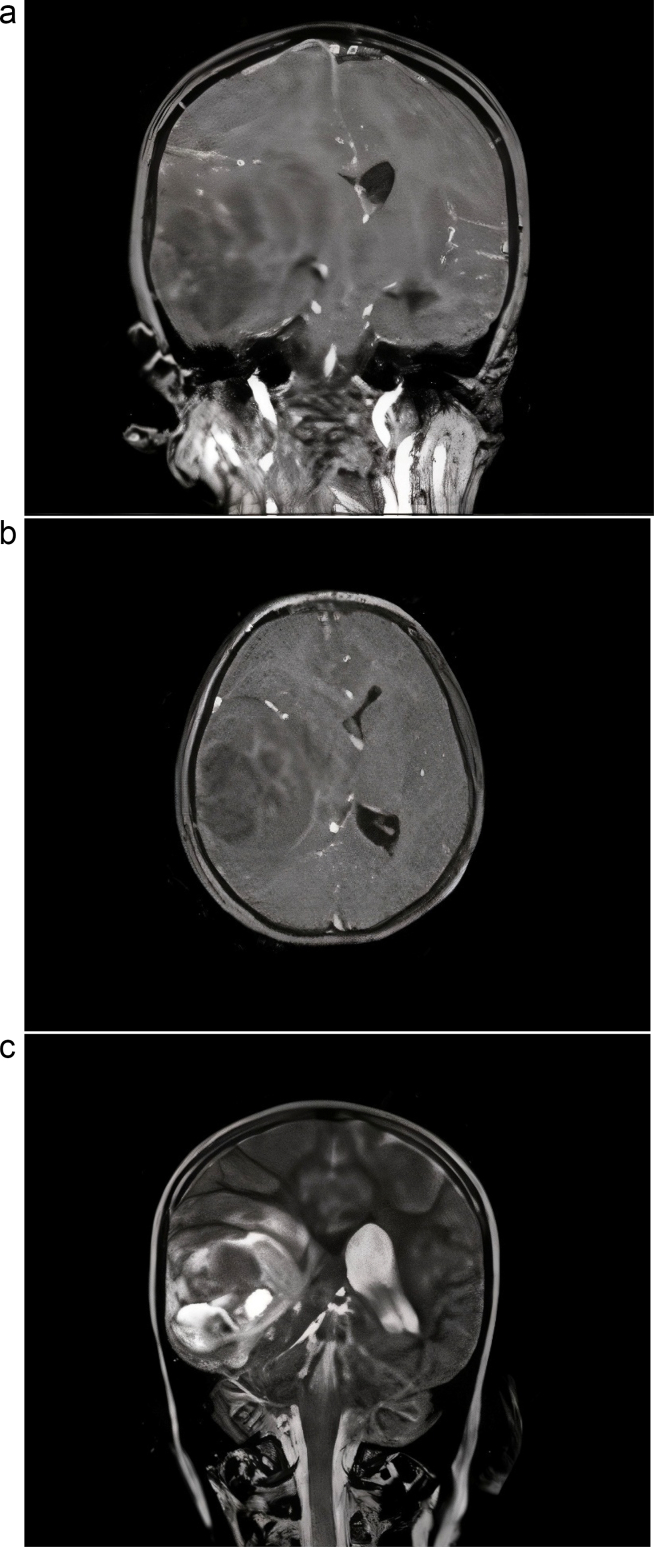
Magnetic resonance imaging at admission time. (a) T1 (coronal view) + gadolinium (contrast) injection. (b) T1 (axial view) + gadolinium (contrast) injection. (c) T2 (coronal view).

**Fig. 2:**
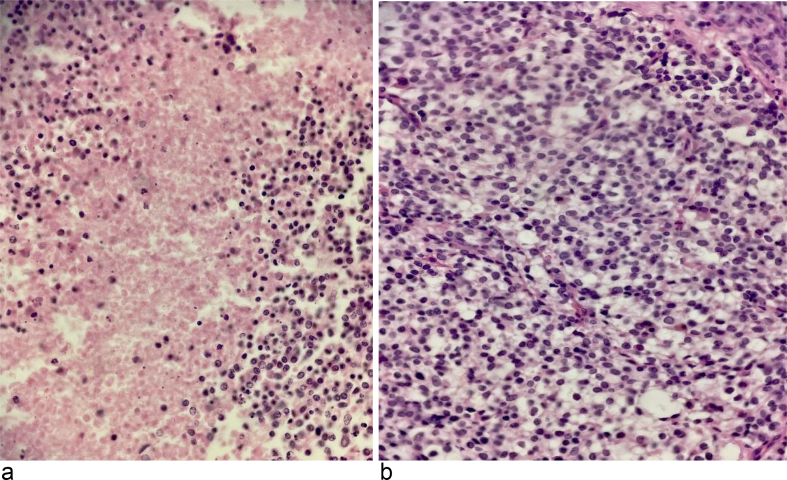
(a) Mitotically active hyper-cellular sheets of densely packed cells with rather small monotonous round nuclei, foci of perinuclear clearing resembling an oligodendroglioma-like appearance, and (b) areas of necrosis. ×40, H&E.

Throughout the course of hospitalization, the patient's overall health progressively declined, marked by an alteration in mental status and a focal seizure episode. Magnetic Resonance Imaging (MRI) of the brain disclosed a prominent mass in the right hemisphere, implicating the right temporal lobe and measuring 5 × 3 × 1 cm (Fig. 1). Subsequently, the patient underwent right temporal craniotomy, resulting in the gross total excision of the tumor. Histopathological examination (Fig. 2) followed by ancillary immunohistochemical and molecular studies, led to the rare diagnosis of scGBM.

## 3 Follow-up

**Fig. 3:**
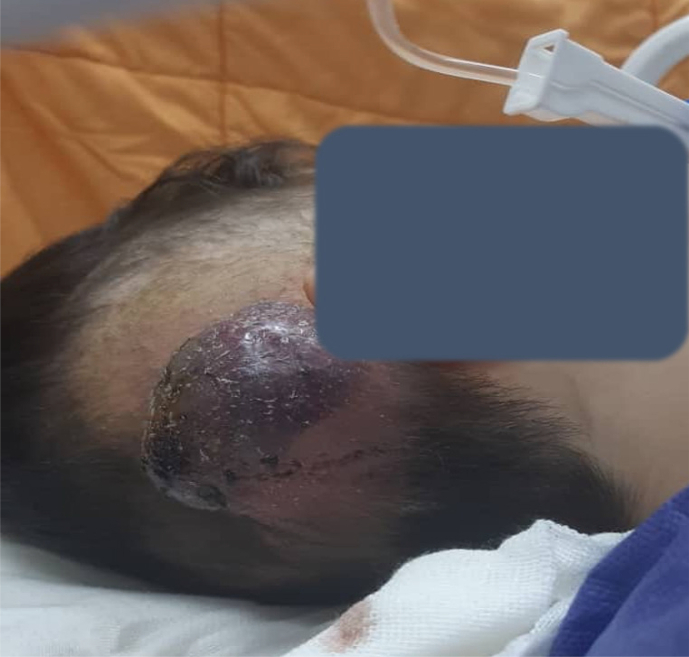
Subcutaneous recurrent tumor at the surgery site, following adjuvant radiotherapy.

**Fig. 4:**
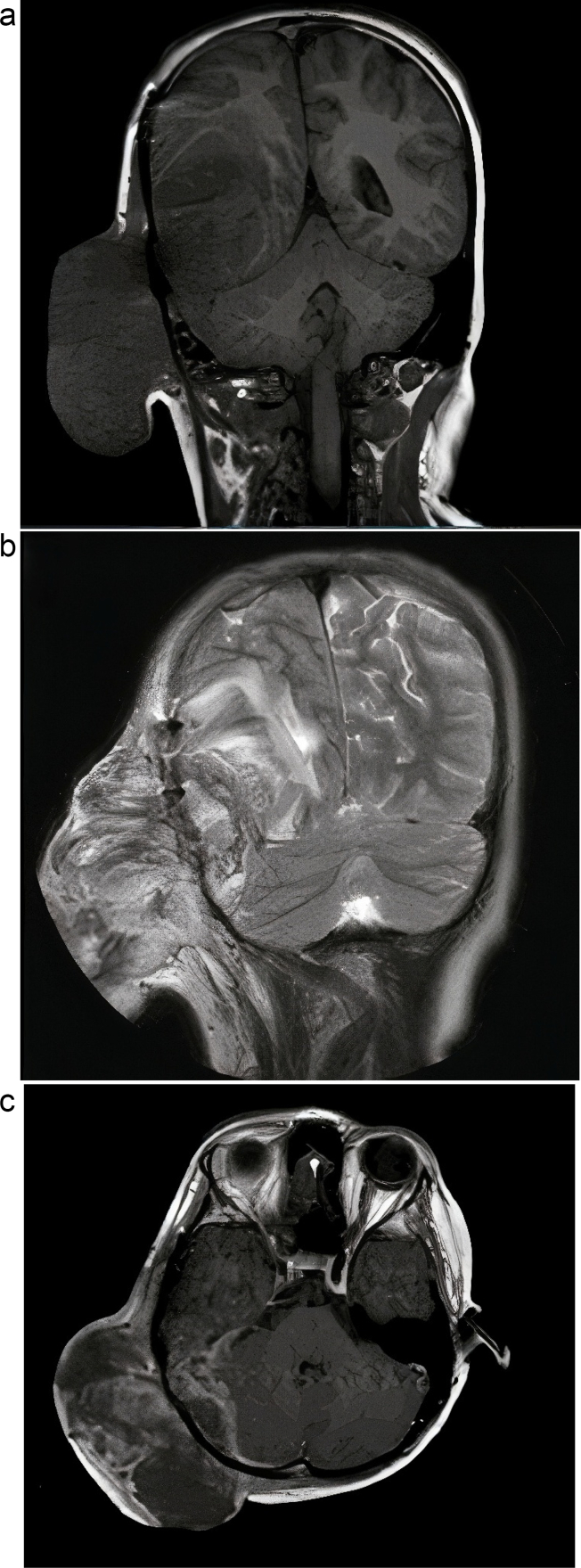
Magnetic resonance imaging following tumor relapse. (a) T1 (coronal view). (b) T2 (coronal view). (c) T1 (axial view) + gadolinium (contrast) injection.

**Fig. 5:**
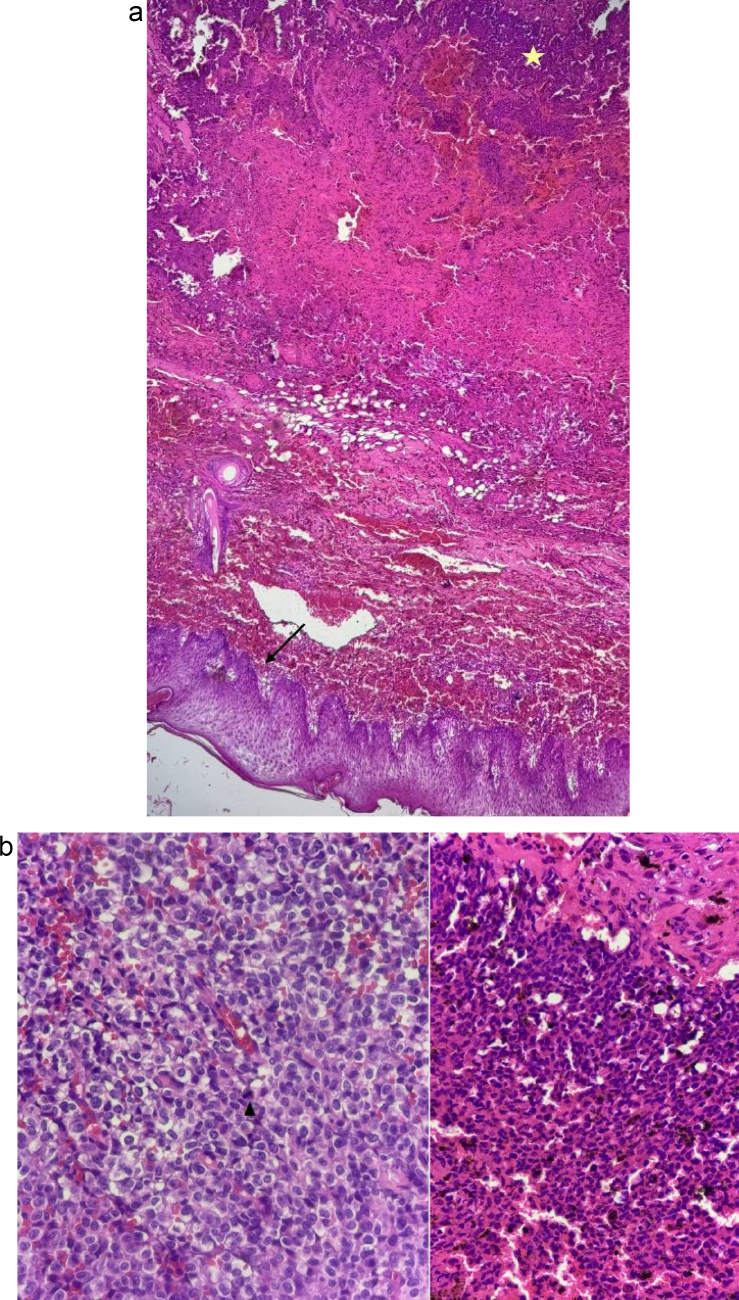
(a). Epidermis/scalp skin (arrow) and infiltrating tumor in subcutaneous tissue (asterisks) with evidence of fresh and chronic hemorrhage. ×4, H&E. (b). Recurrent tumor with scattered mitotic activities (arrowhead) and multiple foci of hemosiderin-pigment deposition. ×40, H&E.

The patient initially underwent surgical resection of a scalp tumor, followed by course of adjuvant radiotherapy. After 32nd radiotherapy session, approximately seven months post-initial surgery, the patient presented with a 7 × 7 cm subcutaneous mass at the previous surgical site in the right parieto-occipital region of the scalp (Fig. 3, Fig. 4), accompanied by overlying skin necrosis, raising suspicions of radio-necrosis. Upon surgical exploration, a substantial mass of tumorous tissue was identified and subsequently excised. Histopathological examination of the specimen confirmed subcutaneous invasion of the recurrent tumor through the craniotomy defect (Fig. 5). Due to the extent of cutaneous necrosis, multiple reconstructive plastic surgeries were performed, ultimately achieving successful restoration. The six-month prognosis following the second surgical operation indicates that the patient remains in stable condition, with no signs of tumor recurrence.

The patient underwent a course of radiotherapy as an adjuvant therapeutic intervention. Following a period of clinical stability, the patient presented with a bulging at the surgical site on the scalp, accompanied by necrosis of the overlying skin, raising suspicions of radionecrosis. Upon surgical exploration, a substantial mass of tumorous tissue was identified and subsequently excised. Due to the extent of cutaneous necrosis, multiple reconstructive plastic surgeries were performed, ultimately achieving successful restoration.

Following the completion of a 32-session course of radiotherapy, the tumor recurred. a subcutaneous mass measuring 7 × 7 cm at the previous surgical site in the right parieto-occipital region of the scalp (Fig. 3, Fig. 4), 7 months post-initial surgical resection. Consequently, the patient underwent a second surgery. Histomorphological study of the specimen confirmed subcutaneous invasion of the recurrent tumor through craniotomy defect (Fig. 5). The 6-month prognosis following the second surgical operation indicates that the patient remains in stable condition, with no signs of tumor recurrence observed at present.

## 4 Histopathological examination

The initial histopathological examination unveiled mitotically active hyper-cellular sheets of densely packed cells with relatively small monotonous round nuclei, foci of perinuclear clearing resembling an oligodendroglioma-like appearance and areas of necrosis (Fig. 2). Consequently, additional investigations were pursued, including complete IHC panel and molecular study according to the differential diagnosis including ependymoma, sarcoma, oligodendroglioma, and lymphoma.

Upon subsequent IHC/molecular investigations, a shift in the diagnostic course emerged. Notably, IHC profiles, inclusive of positive expressions for GFAP, S100, P53, a high KI67 index, and negative results for CK, EMA, and LCA, accompanied by a wild-type IDH status and absence of 1p19q codeletion, supported the ultimate diagnosis of glioblastoma, small cell variant, WHO grade 4.

A notable challenge marked the diagnostic journey, as initial evaluations implied disparate directions, including clinical and radiological findings followed by histomorphological, complementary immunohistochemical (IHC), and molecular studies.

## 5 Discussion

GBM, classified as IDH-wildtype, CNS WHO grade 4, stands as the notable manifestation of primary brain tumors, characterized by an estimated one-year survival rate under current multimodal therapeutic approaches [8,9]. An infrequent pathological variant, scGBM, constitutes a mere 10 % of GBM diagnoses, with an additional 11 % exhibiting similar focal, small-cell-like attributes [10,11]. Despite occasional misinterpretation as high-grade oligodendroglial tumors or lower-grade astrocytomas, scGBM asserts an aggressive nature, aligning with grade 4 gliomas [12]. Although the absence of 1p/19q deletion is a consistent feature of scGBM, the presence of EGFR amplification differentiates it from oligodendroglial tumors [[13], [14], [15]]. Radiologically, scGBM typically parallels conventional GBM, except for a higher incidence of multifocality [4]. Morphologically, scGBM manifests as densely packed, small-sized astrocytes characterized by a rounded to elongated morphology, brisk mitotic activity, and a high nuclear-cytoplasmic ratio [4]. While necrosis and microvascular proliferation may be present to varying degrees, they are not pathognomonic for scGBM, distinguishing it from conventional GBM [10]. The diagnosis of scGBM relies on histopathological assessment; however, IHC may be prudent to eliminate alternative differentials, such as lymphoma, anaplastic oligodendroglioma, primitive neuroectodermal tumor, and metastatic small cell carcinoma. Investigating 1p/19q codeletion by FISH study could also be imperative to exclude anaplastic oligodendroglioma, the most neighboring differential diagnosis [11].

This case report represents an exceedingly rare instance of small-cell glioblastoma occurring in a pediatric male patient, an atypical occurrence given the prevalence of GBM as a prominent adult brain tumor. Age emerges as a significant risk factor, with the median age of GBM patients, particularly those with IDH-wild type, approximating 62 years [16]. Its occurrence in the pediatric demographic is anticipated to be less than 15 %, notwithstanding gliomas constituting one-third of all pediatric brain tumors, with an incidence rate of 5.7 per 100,000 for various pediatric brain tumors [16]. Furthermore, the small-cell variant of glioblastoma manifests at an incidence rate of 5–10 % of all gliomas [17].

The initial histomorphological features of the tumor consist of an oligo-like fried egg appearance, with foci-evoked Ependymoma as the top differential diagnosis. The patient's age further supported this hypothesis, as ependymoma stands as the third most prevalent childhood malignancy of the central nervous system (CNS), and approximately 30 % of all ependymomas occur in teenagers and children, with 12 % of pediatric brain tumors attributed to ependymoma [18]. Notably, 90 % of juvenile ependymomas are intracranial, and one-third of intracranial lesions are supratentorial [18,19]. Anaplastic pediatric oligodendroglioma was considered as the secondary diagnostic consideration. Immunohistochemical examination, however, prompted a deviation from the initial histopathological observations. The absence of IDH-mutation (IDH-wild type) in immunohistochemistry ruled out both primary morphological-based diagnoses, ultimately leading to the rare diagnosis of scGBM (WHO grade 4), confirmed by the molecular study. Other molecular characteristics of scGBM, including TERT promoter mutations, EGFR amplification, or combined gain of chromosome 7 and loss of chromosome 10 (+7/−10) mentioned in the 2021 WHO Classification of CNS Tumors, were not possible to examine [8].

Recent research has highlighted the prognostic importance of these molecular changes in glioblastoma. For example, mutations in the TERT promoter increase telomerase activity, providing tumor cells a growth benefit and consistently linking to reduced overall survival. Similarly, EGFR amplification intensifies oncogenic signaling pathways that facilitate cell proliferation and resistance to typical therapies, thus acting as a marker of aggressive tumor behavior. Furthermore, the simultaneous gain of chromosome 7 and loss of chromosome 10 (+7/−10) signifies a condition of severe genomic instability, which is closely linked with an unfavorable prognosis [8,20].

The observed overlap in histopathological patterns between the initially suggested and the final diagnoses is noteworthy, presenting a pivotal consideration. The histomorphological appearance, predominantly steering our diagnostic approach, which appeared to be deceptive, underscores the significance of ancillary studies, including immunohistochemical and molecular investigations, in refining diagnostic certainty [21].

Given its rarity, there are no standardized treatment protocols for scGBM, forcing adherence to regimens employed for classical GBM. Studies by Huang et al. and Takeuchi et al. reveal comparable median survival rates for GBM and scGBM patients despite the latter receiving higher radiotherapy doses [17,22]. Prognoses, however, remain unfavorable, with aggressive behavior evidenced by limited overall survival, ranging from 5 to 23 months [17,22].

## 6 Conclusion

This case report illustrates the diagnostic and therapeutic challenges posed by small cell glioblastoma (scGBM) in pediatric patients, a context complicated by the tumor's rarity and its symptomatic overlap with other central nervous system (CNS) malignancies. Despite the initial presentation, which was indicative of other CNS tumors, comprehensive immunohistochemical and molecular profiling proved essential for the accurate diagnosis of scGBM. The report underscores the aggressive nature and dismal prognosis of scGBM, which, despite its unique histological characteristics and the younger demographic of the patient, closely mirrors those of traditional glioblastoma (GBM). The lack of standard treatment protocols for scGBM underscores the necessity of customizing therapeutic approaches based on general GBM treatment frameworks, highlighting an urgent need for further research and specific guidelines applicable to this variant. The contribution of this case study is significant, enriching the sparse literature on pediatric scGBM and underscoring the critical importance of exacting diagnostic methodologies to inform tailored treatment plans (Table 1).

**Table 1:** Summary of all SCGBM diagnosis case reports searched in PubMed in the last five years with the keywords: small cell AND glioblastoma.

Article title	Author's name	Case(s)
Small cell glioblastoma multiforme: a case series and clinicopathological update [4]	Yadav et al.	1. A 56-year-old man with headache and altered sensorium. 2. A 57-year-old man with headache, vomiting, and right-sided weakness. 3. A 62-year-old man with a headache. 4. A 53-year-old man with headache, nausea, and memory decline. 5. A 36-year-old woman with headache and vomiting.
Dramatic clinical response in the treatment of small cell glioblastoma multiforme [6]	Zaman et al.	A 51-year-old woman with right lower limb weakness, decreased mobility, imbalance, and memory disturbance. There was a history of a malignant ependymoma in 5th year of life.
A case report of preoperative and postoperative 7.0 T brain MRI in a patient with a small cell glioblastoma [23]	Paek et al.	A 45-year-old woman with a one-month headache history and left hemiparesis.
Small cell glioblastoma of the Sella turcica region: a case report and review of the literature [1]	Deng et al.	A 42-year-old woman with amenorrhea and lactation, diabetes insipidus, and elevated ICP
Differential diagnosis of small cell glioblastoma and anaplastic oligodendroglioma: a case report of an elderly man [5]	Takahashi et al.	A 72-year-old man with falling, dizziness, a history of slow-moving, aphasia, amnesia
Primary spinal cord small-cell glioblastoma: a case report and literature review [24]	Caro-Osorio et al.	A 48-year-old woman with a history of numbness in the left arm and cervical pain
FNA cytology in pediatric small cell glioblastoma [25]	Kalogerak et al.	An 11-year-old boy with symptoms of encephalitis
Conflicting pathology reports: a diagnostic dilemma [26]	Shahar et al.	A 47-year-old man with a 2-day history of progressive right leg weakness
Severe cerebral edema following nivolumab treatment for pediatric glioblastoma: case report [27]	Zhu et al.	A 10-year-old girl with a history of a biallelic PMS2 mismatch repair deficiency, in the first episode, underwent resection of a left frontoparietal glioblastoma, represented by new-onset seizures and mood changes.
Rapid progression of subcutaneous glioblastoma: a case report and literature review [21]	Wang et al.	A 73-year-old woman with postoperative intracranial GBM spreading to the subcutaneous tissue via the channel of craniotomy defect.

## CRediT authorship contribution statement

All authors contributed equally to this work.

## Consent for publication

Written informed consent was obtained from the patient's legal guardian for publication of this case report and any accompanying images. A copy of the written consent is available for review by the Editor-in-chief of this journal.

## Ethical approval

This case report adhered to the ethical principles outlined in The Declaration of Helsinki and received the necessary affirmation from the local research ethics committee.

## Guarantor

Zoheir Reihanian, MD, Associate Professor (correspond author).

## Research registration number

Not applicable.

## Funding

The authors received no financial support for this article's research, authorship, and publication.

## Declaration of competing interest

None declared.

## Data Availability

The data supporting this study is available from the corresponding author upon reasonable request.
